# The Marriage of Faith and Science

**Published:** 1914-05-16

**Authors:** Charles Gardner

**Affiliations:** Chaplain to the Guild of Health. Bushey


*To the Editor of* The Hospital.

### The Marriage of Faith and Science.

*To the Editor of* The Hospital.

Sir,—In your friendly reference to those Churchmen who are interested in spiritual healing, you suggest that they might make their position clearer to the doctors, who recognise the large part that mind plays in all healing, if they would define what they conceive to be the difference between spirit and mind.

Now I should say that we are anxious to separate healing from the mental processes in which it has been entangled by Christian scientists, mental scientists, mental healers, etc., and therefore we have not an immense deal to say about mental, hypnotic, suggestive, or metaphysical therapeutics. While we readily acknowledge that the mind acts on the body, and the body on the mind, we prefer to emphasise the truth that life is at war with disease and death, and we wish to give fullest opportunity for the life forces to prevail. Christians are coming to see at last that Christianity is a new life and a new mode of living. Christ said: "I am come that they might have Life, and that they might have it more abundantly."

When we turn to the past we find that Christians have set up an artificial division in themselves and said this part is soul, this mind, this spirit, this body. In consequence, when they read the Gospel, with its promise of Life, they accepted the Life of their "immortal souls," and quietly ignored their "vile bodies." Now the whole trend of modern thinking is to regard man as a unit, and so to realise William Blake's contention that body is that part of the soul discerned by the five senses. In so far as Christians have realised their unity, they have claimed Life for their whole being; and they have found as a matter of experience that as they have become more and more receptive to the spirit of life, so their bodies have been quickened. The enormous increase of the life force has resisted disease and maintained health, and when there has been disease the struggling life has been reinforced in a thousand instances sufficiently to restore to health.

But what else are the doctors aiming at? All their means are used to give the healing life force full play. Therefore there is no antagonism between the spiritual and medical healer. The doctor clears away all obstructions to the working of the life energy, the spiritual healer intensifies the life energy itself, and if both will work together the results will be such as the world has never yet seen.—I am, yours faithfully,

Charles Gardner,
Chaplain to the Guild of Health.

Bushey, May 9, 1914.

